# The Criminal Law Amendment Bill

**Published:** 1917-03-31

**Authors:** Alison Neilans

**Affiliations:** *Secretary*. The Association for Moral and Social Hygiene, 19 Tothill Street, Westminster, S.W. 1


### THE EDITOR'S LETTER-BOX.

### The Criminal Law Amendment Bill.

*To the Editor of* The Hospital.

Sir,—I have only just seen your criticism on Dr. Helen Wilson's attitude to the Criminal Law Amendment Bill, and I think it is probably made under a misapprehension.

Dr. Wilson's article, which appeared in the *Times* on the Monday following the introduction of the Bill, had been written several days, but at the *Times* office on the Sunday she saw the Bill for the first time, and was asked to introduce a paragraph criticising it. You will, I think, recognise that Dr. Wilson was not blind to the defects of Clause 1 by a perusal of the letter (a copy of which I enclose), which is signed by Dr. Wilson, our chairman, and myself.

With reference to your further criticism as to Dr. Wilson's conclusion that Clause 2 was specially aimed at prostitutes, I think you must have overlooked the fact that the clause would be in practice only applicable to persons convicted of offences in the Schedule of the Bill, and of those offences more than four-fifths would be women convicted of solicitation. I venture to think, therefore, that Dr. Wilson was perfectly correct in her statement "it is difficult to avoid the conclusion that the Bill is aimed at prostitutes."

I am not authorised by Dr. Wilson to answer your comments, which she possibly has not seen, but I think it only right that you should do Dr. Wilson justice in the matter.—Yours faithfully,

Alison Neilans, *Secretary*.

The Association for Moral and Social Hygiene,
19 Tothill Street, Westminster, S.W. 1,
March 19, 1917.

### Copy of Letter Signed by Dr. Helen Wilson and the Chairman and Secretary of the Association for Moral and Social Hygiene.

CRIMINAL LAW AMENDMENT ACT.

